# Influence of heat treatment processes on microstructure evolution, tensile and tribological properties of Ti6Al4V alloy

**DOI:** 10.1038/s41598-023-38250-2

**Published:** 2023-07-12

**Authors:** Ramadan N. Elshaer, Shimaa El-Hadad, Adel Nofal

**Affiliations:** 1 Tabbin Institute for Metallurgical Studies, Cairo, Egypt; 2 Central Metallurgical Research and Development Institute, Box 87, Helwan, Egypt

**Keywords:** Engineering, Materials science

## Abstract

The influence of heat treatment processes on microstructure, tensile and tribological properties of Ti6Al4V alloy was investigated. The specimens were heated for 30 min at 925 °C and then cooled at various rates by water quenching, air cooling, and furnace cooling. After that, the samples were aged for four hours at 600 °C. Three phases make up the microstructure: primary α-phase (α_p_), secondary α-phase (α_s_), and retained β-phase (β_r_). Cooling in the air and water followed by aging (AC + Aging and WQ + Aging) resulted, α_s_-phase precipitating inside β_r_-phase. The highest hardness of 35 HRC was recorded for WQ + Aging specimen due to existence of a high amount of β_r_-phase and precipitation of α_s_-phase. On the other hand, the lowest hardness of 26 HRC was obtained for the FC specimen. AC specimen achieved the highest elongation value of 14%. However, WQ + Aging specimen exhibited the highest ultimate tensile strength of 1028 MPa. For WQ + Aging and AC + Aging specimens, the ideal balance of strength and elongation was discovered. The wear resistance of solution-treated specimens was significantly improved by the aging process and 125% improvement could be achieved in WQ compared to WQ + Aging specimens.

## Introduction

Titanium and its alloys are well-known and used in various industries including aircraft, military, and petrochemicals due to their desirable features such as high specific strength, resistance to corrosion, and high temperatures. Ti6Al4V is the most common two-phase (α + β) titanium alloy and is currently the most extensively used titanium alloys. Due to its many advantageous characteristics, such as its strength-to-weight ratio, biocompatibility, exceptional corrosion resistance, and processability^1–4^, Ti6Al4V alloy is frequently utilized in the medical device sector and medical implant industries^5, 6^. It is also widely utilized in aerospace and aviation industries for structural and engine parts like fan blades, discs, and shafts because of its remarkable mix of strength and ductility. Accounts for nearly 60% of global titanium production and more than 50% in the USA^7, 8^. Another characteristic of Ti6Al4V that makes it a very well-liked titanium alloy is the ability to produce balanced mechanical properties depending on the application by modifying its microstructure through specific heat treatments^9^. Due to the alloying of 6% Al stabilizing the α-phase and 4% V stabilizing the β-phase, it is Ti6Al4V alloy that keeps both the low-temperature and the high-temperature allotropes at ambient temperature. Therefore, further strengthening of this alloy will have a significant effect.

Solution treatment and aging processes are the conventional heat treatments for Ti6Al4V alloy. Heat treatments such as mill annealing or solution treatment and aging (STA) can be used to alter the mechanical characteristics of Ti6Al4V alloy^10^. STA typically results in 20% increase in tensile strength over mill-annealing^1^. STA is also utilized to enhance the mechanical properties of aerospace elements since it provides adequate ductility. By modifying the microstructure through heat treatment, usually at temperatures in the dual α-β phase area, the tensile and fatigue properties of the Ti6Al4V alloy are improved. The complicated microstructure of the Ti6Al4V alloy has a significant impact on its tensile and fatigue characteristics. The size and volume percentage of the α and β-phases of titanium alloys are frequently used to define their microstructure. Equiaxed microstructure (a uniform structure consisting of equiaxed β grains and grain boundaries of α-phase^11, 12^), which results from recrystallization and globalization process^13^, and lamellar microstructure (with a larger α/β surface area and more directed colonies), which is generated upon cooling from the β phase field, are the two extreme instances of phase arrangements. According to earlier studies, equiaxed microstructure, as opposed to lamellar microstructure, shows the best resistance to fatigue initiation but the lowest resistance to fatigue propagation^14^. Compared to equiaxed microstructure, lamellar microstructure exhibits lower strength, worse ductility, and better resistance to fatigue propagation^15, 16^.

Earlier studies on the microstructure and mechanical characteristics of Ti6Al4V were conducted under varied cooling rates during the solution treatment stage of STA^1, 6, 14–22^. Studies examining the impacts of cooling rate during solution treatment have shown that WQ following isothermal holding causes the change of β-phase to α'-phase, while slower, more gradual cooling causes the production of coarser globular and the change of β-phase to α + β lamellar, which in turn causes the hardness to diminish^18, 19^. Additionally, following WQ plus aging, α' turns into α + β^1, 8^, whereas, after relatively slow cooling, each produced lamella becomes considerably coarser^21–23^. In a similar vein, when using a reduced cooling rate during solution treatment, the hardness after aging is lower^23^. According to Elshaer and Ebrahim^24^ and Morita et al.^25^, water-quenched TC21 and Ti6Al4V alloys in solution treatment can increase its tensile and yield strengths by precipitation of fine α-phase during aging. By studying Ti6Al4V alloy that was air-cooled in solution treatment and then aged at various temperatures, Lin et al.^22^ discovered that when the aging temperature increases, the secondary phases develop and extend into the equiaxed α-phase, then merge to produce a new curved lamellar phase. According to the cooling conditions (WQ and AC) during solution treatment, Pinke et al.^23^ stated phase transformations and hardness changes after STA heat treatment, however, there is inadequate justification for the relationship between the hardness change and the microstructure. Ren et al.^19^ and Gupta et al.^17^ examined microstructure and mechanical characteristics in terms of the cooling rates of solution treatment in STA heat treatment, as WQ vs AC and WQ vs AC vs FC, respectively. But in these experiments^17, 23^, the specimens were only examined after the subsequent aging stage, not right away after solution treatment. As a result, the goal of this work was to apply three different cooling rates to te as-annealed Ti6Al4V alloy, followed by the aging to optimize hardness, tensile and tribological properties.

## Materials and experimental procedures

### Material

Bar-shaped Ti6Al4V alloy (Ø10 × 123 mm) in ASTM F136 Grade 5 Standard. This alloy fabricated via vacuum arc remelting (VAR) and rotary forging was obtained from BAOJI XUHE TITANIUM METAL CO., LTD. The chemical composition of Ti6Al4V was (wt, %): Ti–6.2Al–4V and other minor alloying elements, such as 0.15 max Fe, 0.08 max C, 0.07 max O, 0.05 max N, and 0.012 max H. To reduce the residual stress during hot-forged, stress relief annealing was applied at 550 °C for 2 h and then furnace cooling. A horizontal pushrod dilatometer with computer control (LINSEIS DIL L76 instrument, Germany) was used to measure the transformation temperatures during continuous heating and cooling. Using a wire electrical discharge machine (EDM), a specimen with a diameter of 5 mm and a length of 20 mm was carefully machined for a dilatation test. The specimen was held in contact with the pushrod and heated in static air at a rate of 10 °C/min to 1100 °C before cooling in air to room temperature. WIN-DIL software was used to record the change in specimen length as a function of temperature. The α + β ↔ β phase transformation for as-annealed condition was calculated from the first derivative using an equation between temperature and length.

### Experimental procedures

**Figure 1:**
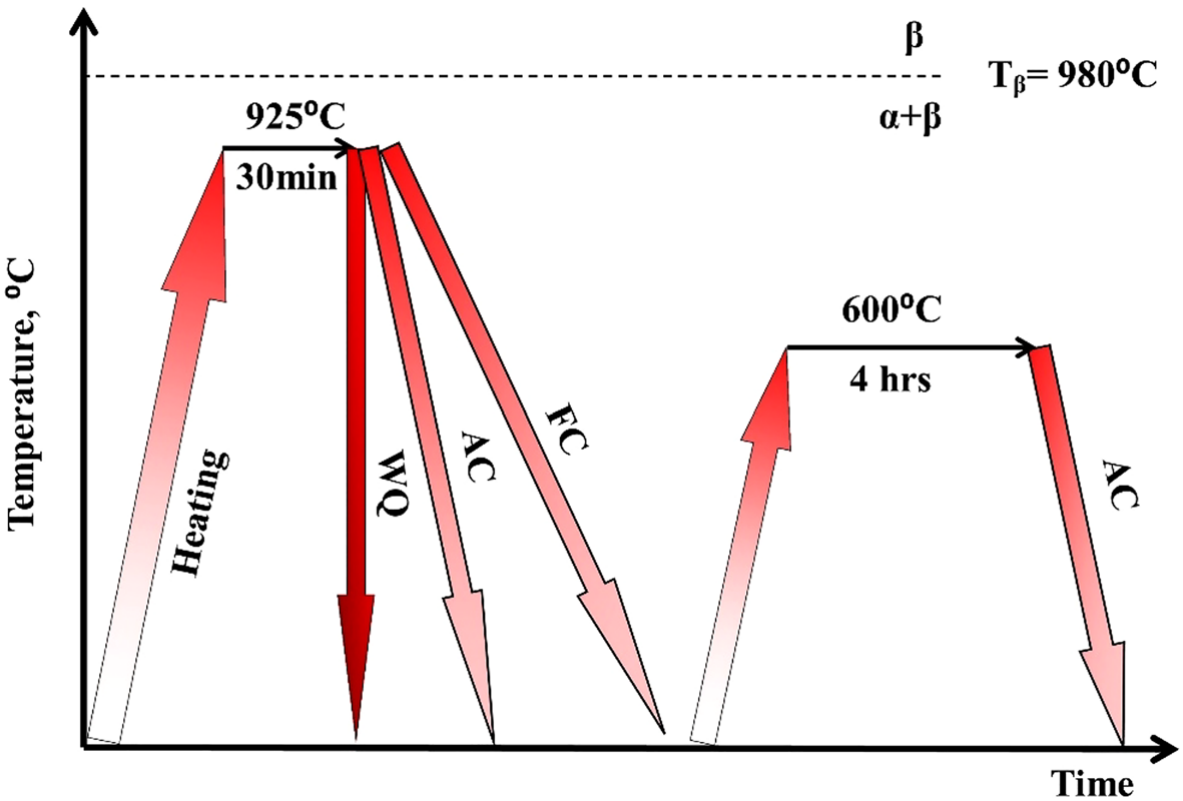
Schematic cycles of heat treatment processes applied to Ti6Al4V alloy.

The solution treatment temperature was determined after finding the transformation temperature (T_β_) for Ti6Al4V alloy. All specimens were solution treated at 925 °C (α-β region) for 30 min, and then cooled to ambient temperature using different rates: (1) furnace cooling (FC), (2) air cooling (AC), and (3) water quenching (WQ). The specimens were then aged for 4 h at 600 °C, followed by air cooling (see Fig. 1). Standard techniques for grinding, polishing, and etching specimens with a combination of 3 ml HF, 30 ml HNO_3_, and 67 ml H_2_O were used to prepare the specimens for metallography investigation. The microstructure evaluation was examined using field emission scanning electron microscope (FESEM) Quanta-FEI/FEG-250 Netherland with Energy Dispersive X-Ray Analysis (EDX). In order to identify the phases, X-ray diffraction with a diffractometer was used. Cu Kα1 radiation was utilized with 2 range of 30°–120° and data were recorded at 0.02 degrees with steps moving at a speed rate of 0.004 degree/min. Image J and Gwyddion softwares have been used for processing the photographs to determine the volume fraction of different phases.

The hardness of the specimens was evaluated using a Rockwell hardness tester. An average of seven readings were taken from each specimen and recorded. Smooth-bar tensile specimens were machined after the heat treatment procedures. Tensile testing was performed on specimens with a gauge length of 30 mm and a diameter of 6 mm according to ASTM E9-16 standard. The tensile test was carried out at room temperature with a crosshead speed of 2 mm/min using a universal testing machine (WDW-300 KN, China). The average of three different tests was used to get data on all tensile properties. FESEM was utilized to look at the fractographic characteristics of several chosen tensile specimens to understand the fracture mechanics of the researched Ti6Al4V alloy. Pin-on-disk wear tribometer testing device (T-01M) was used to measure the resistance to wear at room temperature. The pin specimen with a cylindrical form of 10 × 10 mm was pushed against a rotating tool steel disk (65 HRC). The wear test was performed at sliding speed of 0.2 m/s, normal load of 80 N, time of 15 min and constant sliding distance of 150 m. Before and after the test, the specimens were cleaned and weighed using an electronic scale with 0.1 mg accuracy. The average wear rate during three tests was computed for each condition. The test results were evaluated according to the loss in weight. The wear rate (Wr) was expressed by:

1 $${\text{Wr}} = \frac{{\Delta {\text{w}}}}{{\text{t}}}\;{\text{g}}/{\text{min}}$$

where, Δw: weight loss in grams (g) and t: time in minutes (min)

To calculate the friction coefficient, which is the friction force divided by the applied force (80 N), the value of the friction force was measured during the test by a data acquisition system saved in a PC. The worn surface texture of some selected specimens was seen using FESEM micrographs. The chemical composition and mechanical properties of Ti6Al4V alloy are listed in. Table 1^26^.

**Table 1:** Chemical composition and mechanical properties of Ti6Al4V alloy ^26^.

Chemical composition, wt. %	T_β_, ºC	Hardness, HV	Young's modulus, GPa	Yield strength, MPa	Ultimate tensile strength, MPa	Elongation, %
Al: 5.5–6.7 V: 3.5–4.5 Fe: 0.25 C: 0.2	995	300–400	110–140	800–1100	900–1200	13–16

## Results and discussion

### Dilatometer behavior

**Figure 2:**
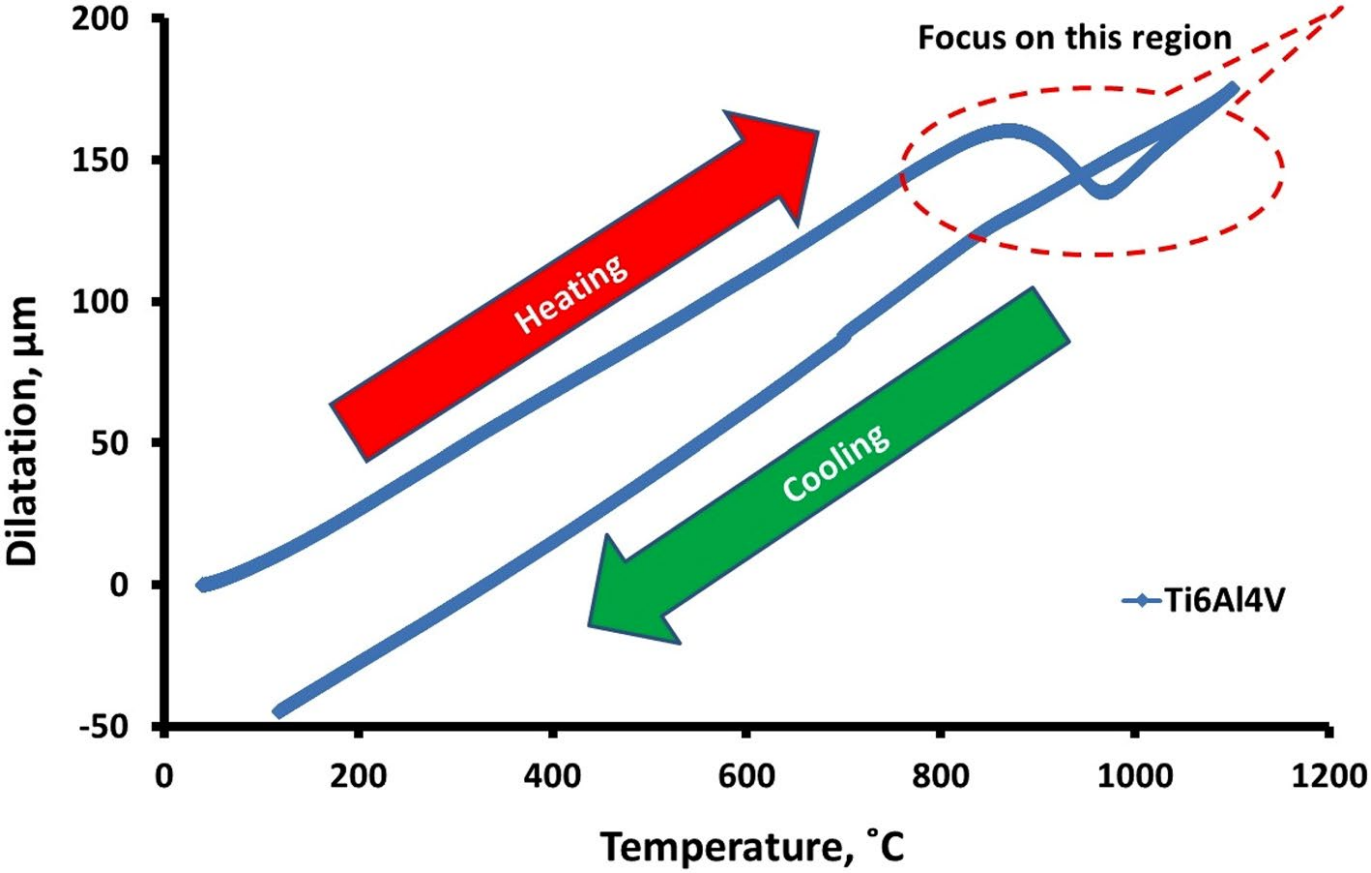
Dilatometric curve of Ti6Al4V alloy (general view).

According to the ASM handbook^27^, the beta transus temperature (T_β_) of Ti6Al4V alloy is 1000 ± 20 °C, with the actual temperature varying depending on the quantity of alloying elements. The general dilatometric curve (temperature-dependent compression/dilatation) was acquired during the continuous heating and cooling of Ti6Al4V specimen, see Fig. 2. The slope changes in the curve that corresponds to changes in specimen length are used to calculate transformation temperatures.

**Figure 3:**
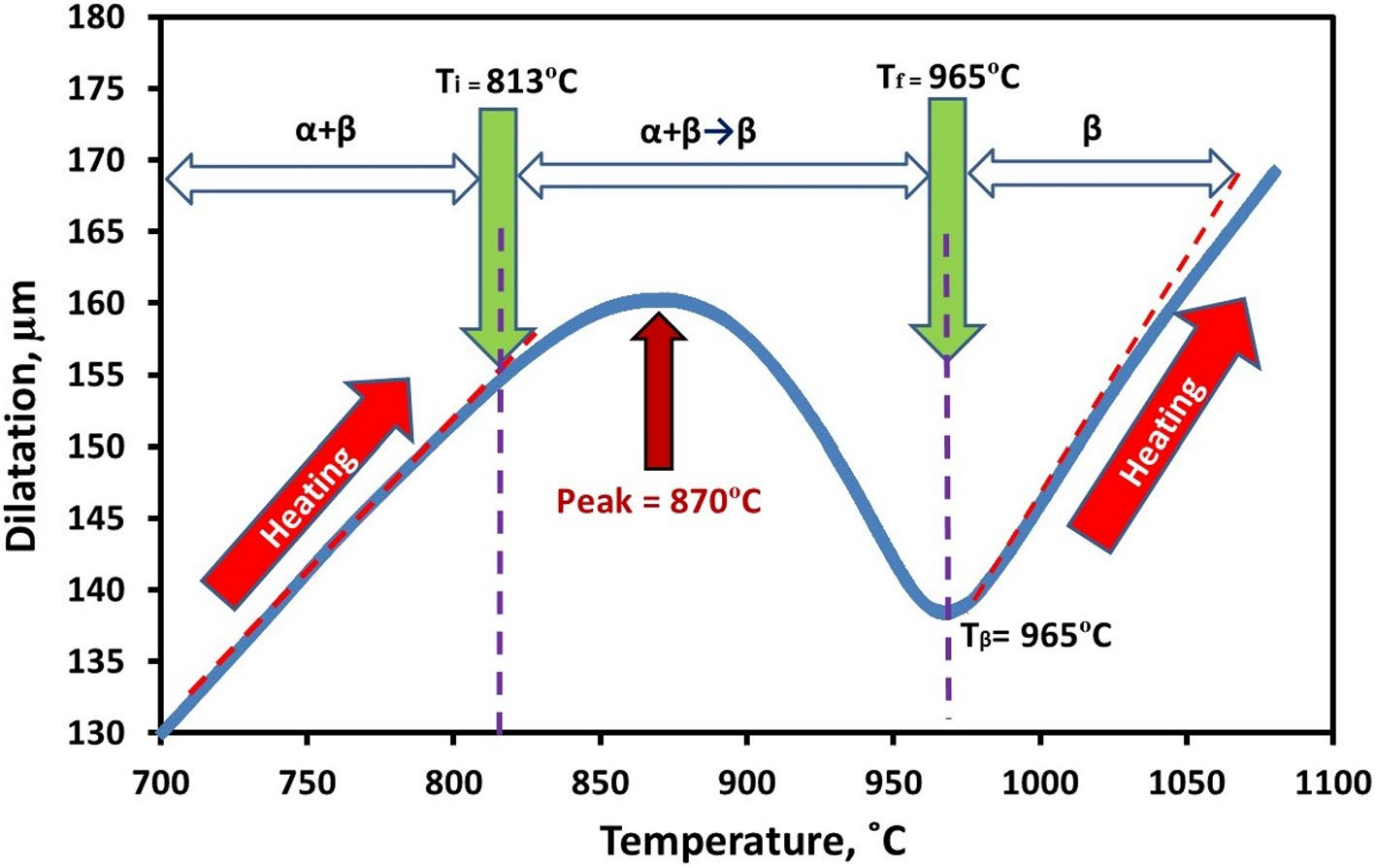
Dilatometric heating curve for α + β → β phase transformation's initial and final temperatures.

The curve became non-linear when the specimen was heated above 813 °C (Fig. 3). In this scenario, the curve reached 870 °C before descending to 965 °C. This might be explained by the shrinking effect generated by the crystal structure upon changing from a duplex (α + β) to a single β-phase structure^28, 29^. As α + β → β phase transformation continues, the specimen continues to shrink until it reaches 965 °C, see Fig. 3. With the rising temperature, the specimen length begins to increase. The curve becomes linear as α + β → β phase transformation is stopped. The specimen continues to shrink while α + β → β phase transformation continues, eventually stopping at 965 °C, as seen in Fig. 3. The length of the specimen rises as the temperature rises, eventually becoming linear once the α + β → β phase change is stopped.

The amount of α-phase reached its maximum at 870 °C (certain peak in Fig. 3). At this temperature, there will be an equilibrium condition between α and β-phases. The equilibrium will be disturbed when the temperature rises above 870 °C, and the α-phase will be converted to β-phase. Then, as the ratio of β to α rises, the phase's expansion takes precedence over the alloy's overall expansion. Consequently, a relative rise in specimen length will be observed on the dilatometric curve until T_β_ (965 °C). A full β-phase occur if the temperature is raised over transus temperature, T_β_ (965 °C). Two distinct reflection points can be seen on the above-mentioned dilatometric curve with rising heating temperature. They are denoted by the letters (T_i_) and (T_f_), which stand for the initial transformation temperature of α + β → β and the final transformation temperature, respectively. T_s_ was measured at 813 °C for the examined Ti6Al4V alloy, while T_f_ was measured at 965 °C.

**Figure 4:**
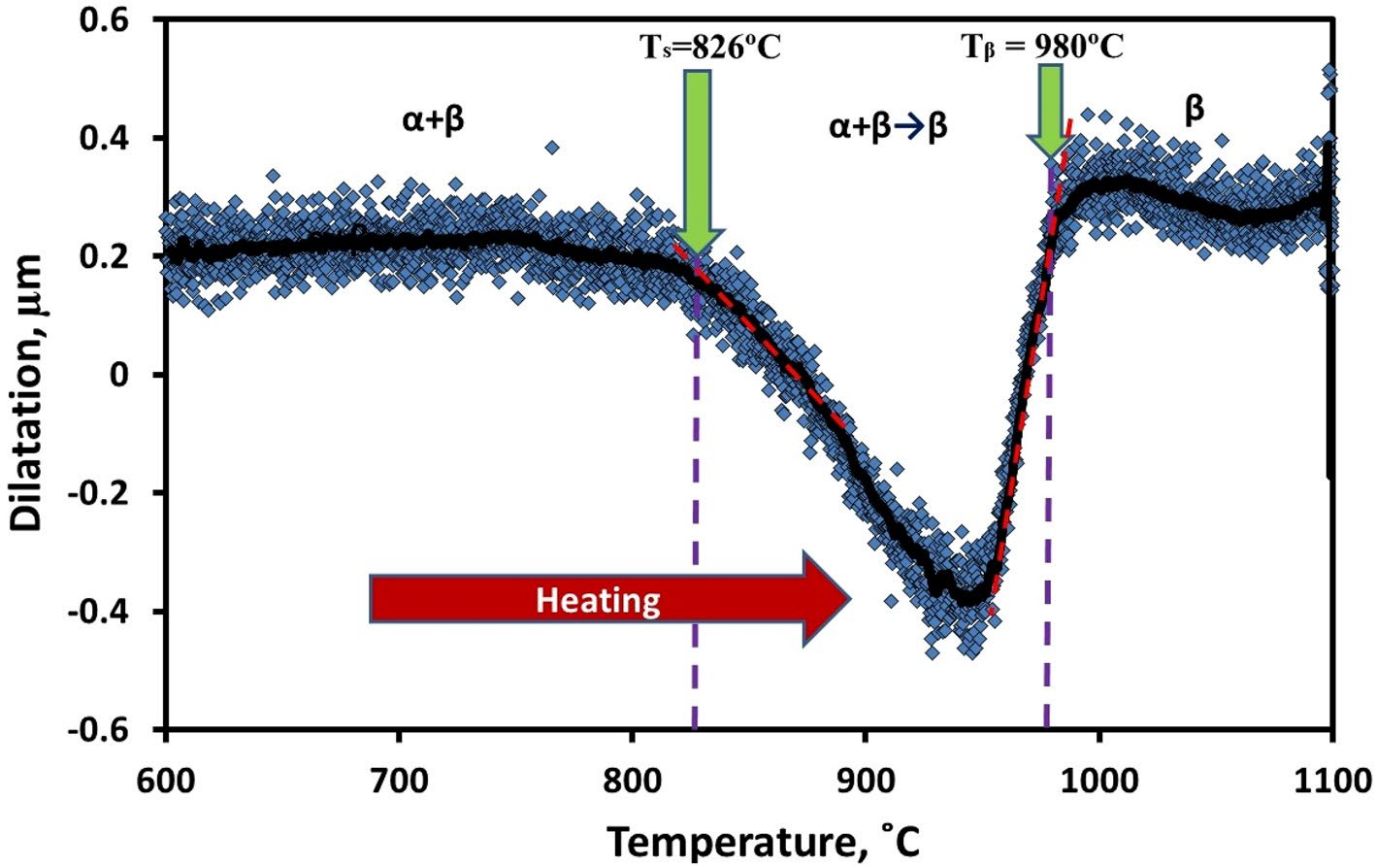
First derivative of the dilatometric heating curve of Ti6Al4V alloy.

Furthermore, the first derivative of investigated dilatometric heating curve revealed that α + β → β transformation's starting and finishing temperatures were more precisely and objectively, Fig. 4. Using the first derivative curve, Fig. 4, the began and finished transformation temperatures were 826 and 980 °C, respectively, which are similar to dilatometric heating curve values (813 and 965 °C). Therefore, T_β_ was 980 °C.

### Microstructure evolution

**Figure 5:**
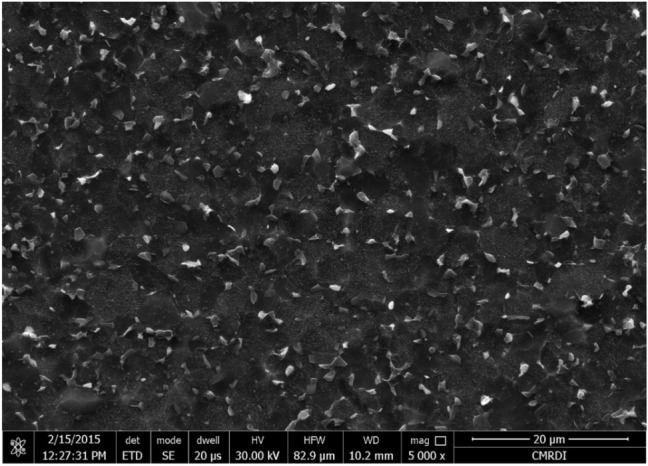
Initial microstructure of as-annealed Ti6Al4V alloy.

**Figure 6:**
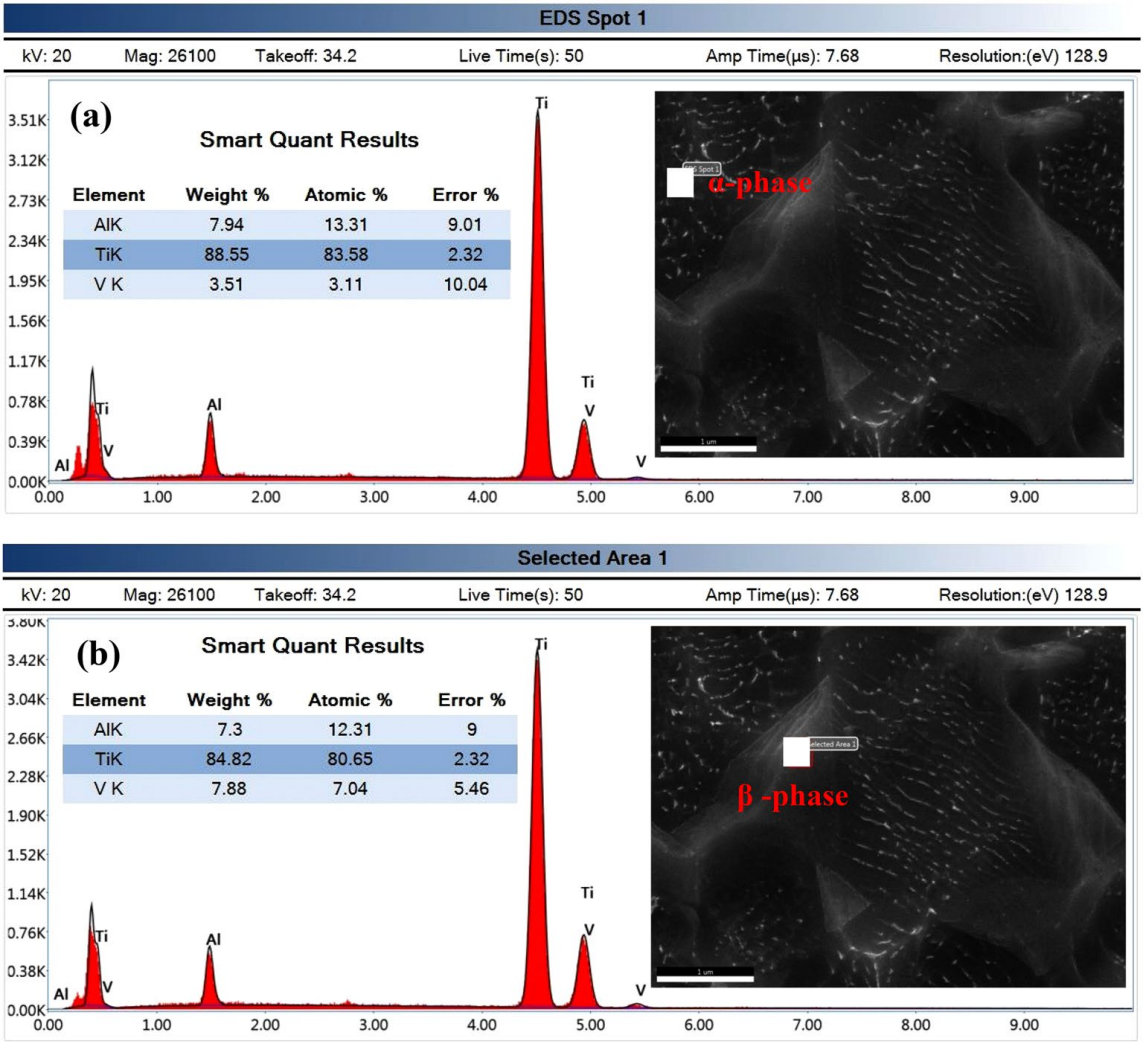
EDX analysis of as-annealed Ti6Al4V specimen: (**a**) α-phase and (**b**) β-phase.

As-annealed Ti6Al4V specimen had an initial microstructure that was equiaxed and comprised a finely equiaxed α phase (α_p_), a black area, and a transformed β phase (β_trans_), a grey area, as shown in Fig. 5. In the β_trans_ phase, the equiaxed α-phase was uniformly distributed. Energy-dispersive X-ray spectroscopy (EDX) was used to investigate both α and β-phases (Fig. 6). A high amount of α-stabilizing element (Al = 7.94%) and a low amount of β-stabilizing element (V = 3.51%) was found in the α-phase, Fig. 6a. However, a high amount of β-stabilizing element (V = 7.88) and a low amount of α-stabilizing element (Al = 7.3%) existed in β-phase, Fig. 6b.

**Figure 7:**
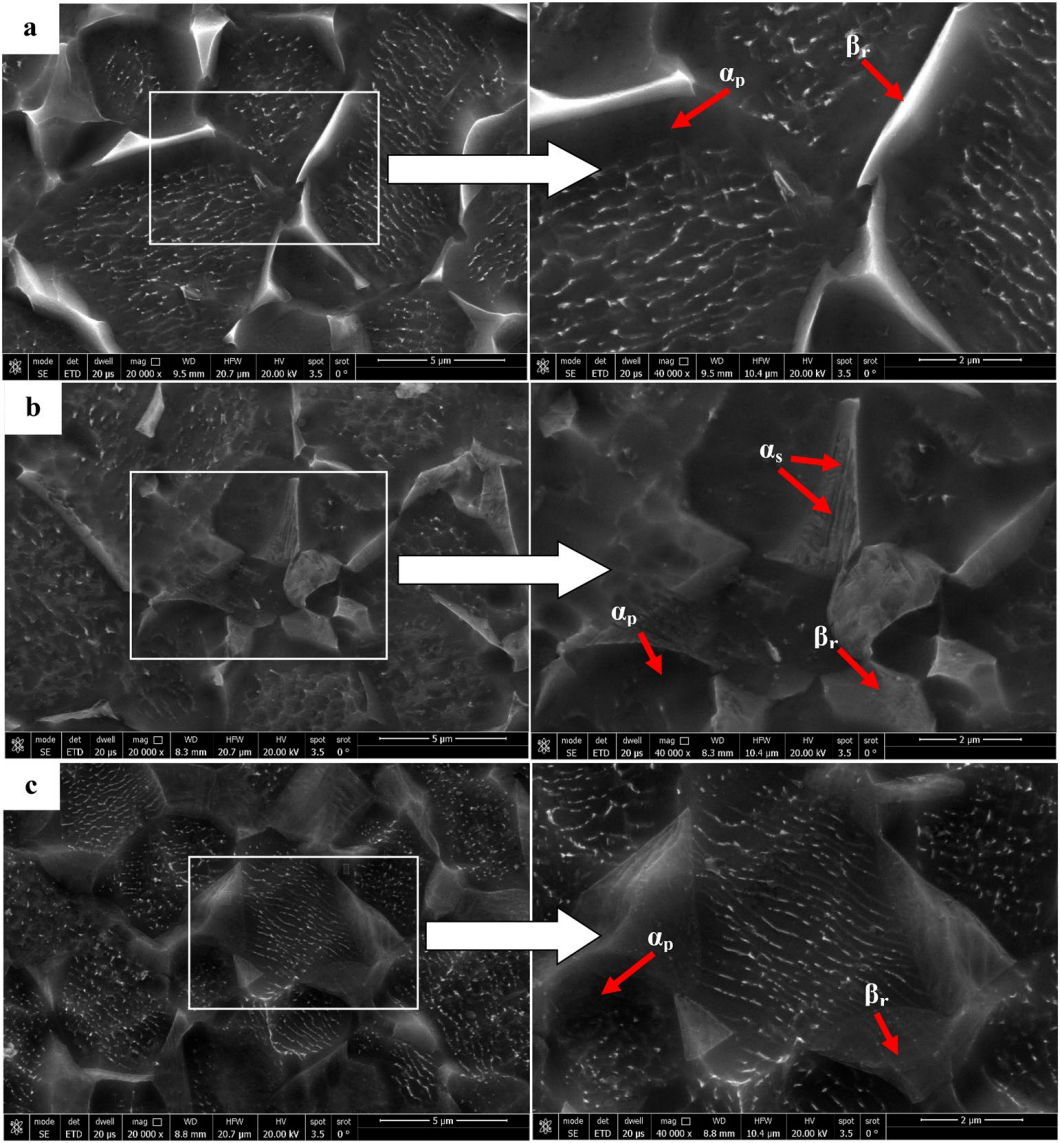
FESEM micrographs for variant cooling rates: (**a**) FC, (**b**) AC, and (**c**) WQ.

**Figure 8:**
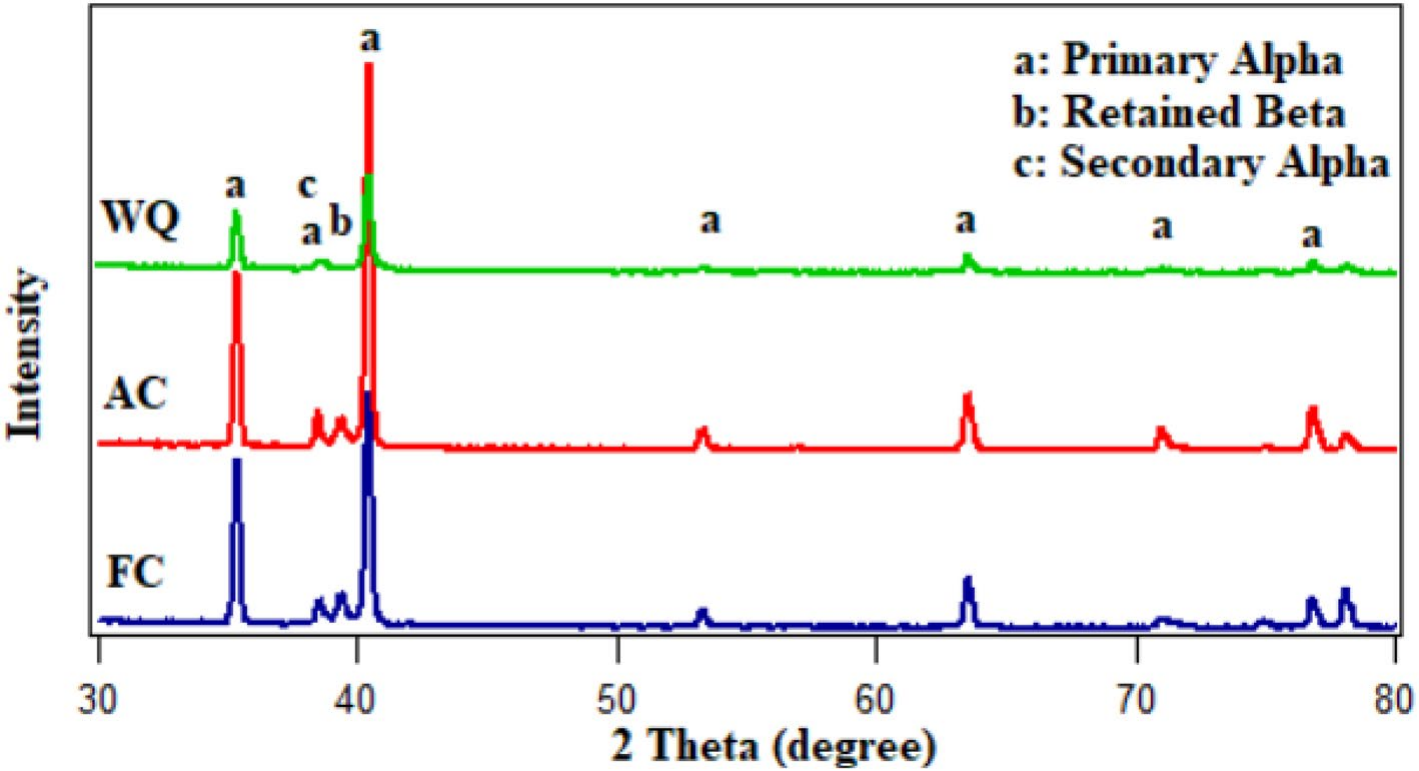
XRD pattern of FC, AC and WQ specimens.

Figure 7 illustrates the microstructure of Ti6Al4V specimens that were heated to 925 °C and then cooled at three distinct rates: furnace cooling (FC), air cooling (AC), and water quenching (WQ). The microstructure of all specimens consists of primary α-phase (α_p_) and retained β-phase (β_r_), with a low amount of secondary α-phase (α_s_) in the case of AC specimen (Fig. 7b). There is no obvious change in the microstructure of FC and WQ specimens except for the volume fraction of α_p_ and β_r_-phases. As seen in Fig. 7a, the very slow cooling rate of FC specimen prevented the α_s_-phase from precipitating because there was not enough driving force to allow the α_s_-phase to form. Naturally, FC specimen didn't produce enough super-saturation, therefore, there wasn't any α_s_-phase precipitation within β_r_-phase^24^. As a result of the β stabilizing elements diffusing inside the β_r_-phase, α_s_-pthe hase did not precipitate in the case of WQ specimens, which led to substantial supersaturation. These factors will afterward stop α_s_-phase precipitation from occurring during β_r_-phase. The possibility of α_s_-phase precipitation within β_r_-phase is therefore eliminated (Fig. 7c). The conclusion is that α_s_-phase precipitated within β_r_ -phase at a medium cooling rate (AC) but not at a slow cooling rate (FC) or high cooling rate (WQ). This observation was supported by Elshaer^11^ work with a difference in the type of titanium alloy but in the same category. Figure 8 displays XRD patterns of the FC, AC and WQ specimens. The patterns confirmed the presence of the phases in the microstructure.

**Figure 9:**
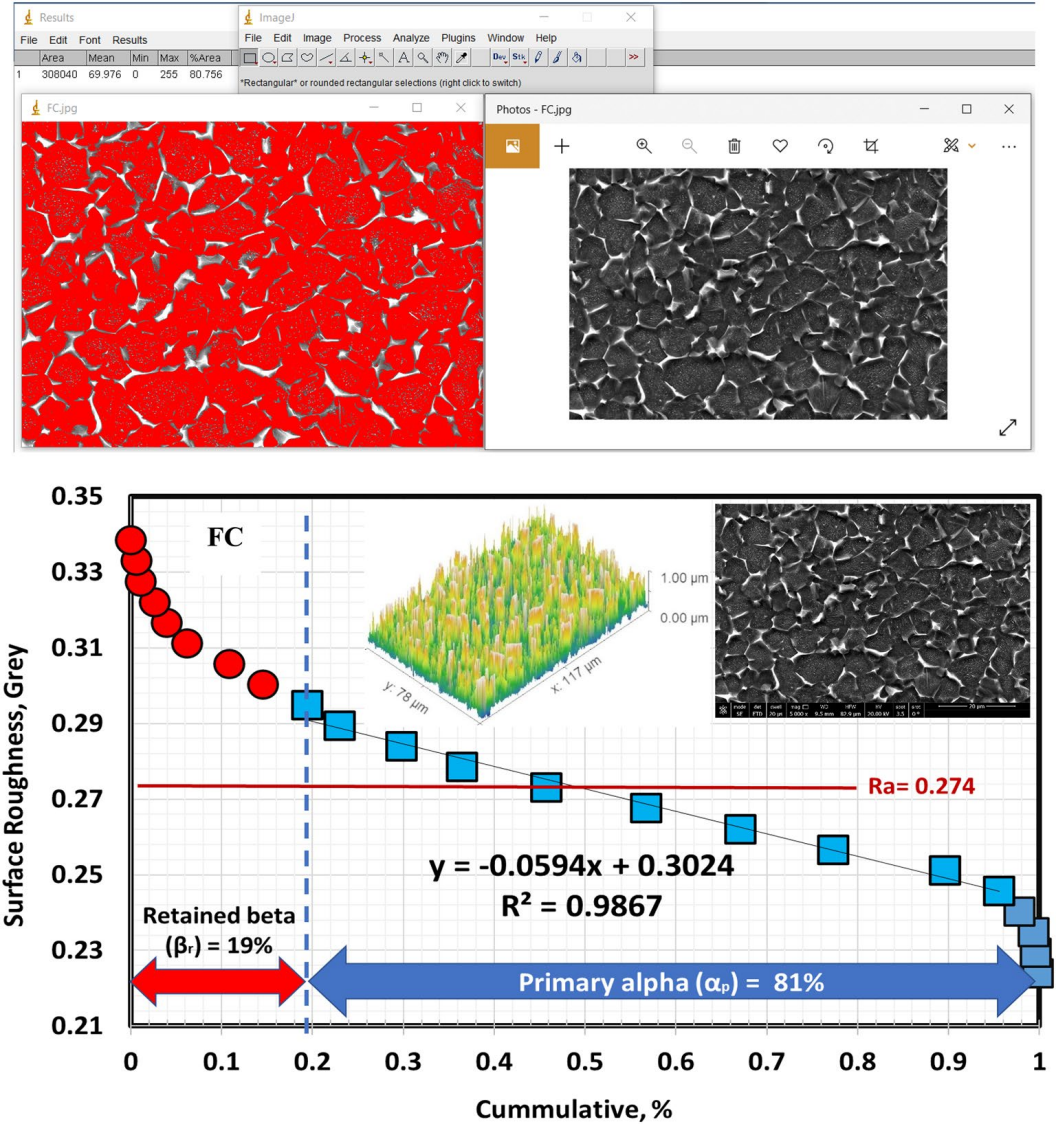
Volume fraction of α_p_-phase for FC specimen.

**Figure 10:**
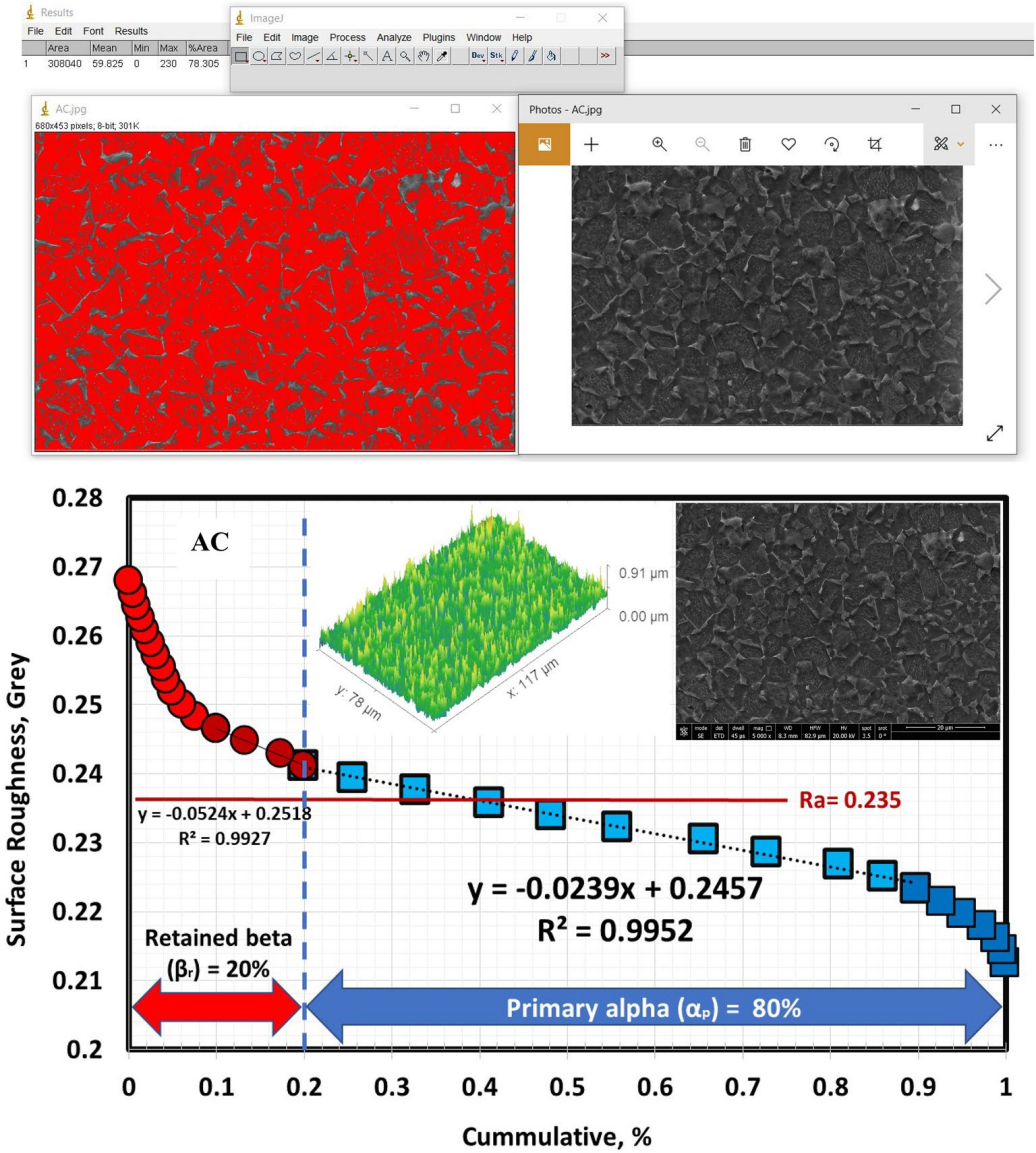
Volume fraction of α_p_-phase for AC specimen.

**Figure 11:**
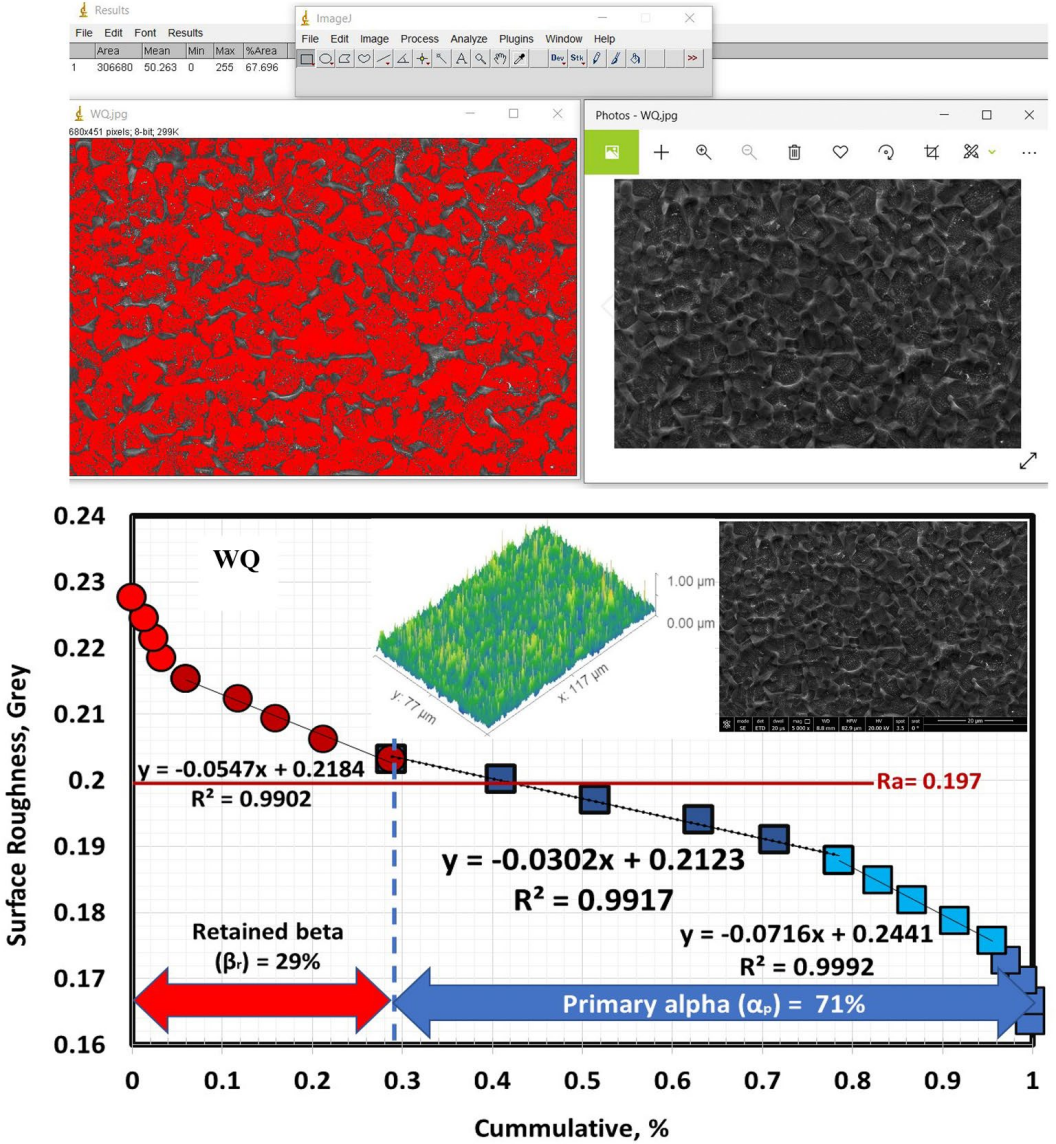
Volume fraction of α_p_-phase for WQ specimen.

According to Figs. 9, 10, and 11, the volume fraction of α_p_-phase generally decreases as the cooling rate increases. The volume fraction of α_p_-phase in the case of FC specimens was calculated to be around 81%. However, 78 and 68% for AC and WQ specimens. As seen in Fig. 8, FC specimens demonstrated an increase in α_p_-phase grain size when compared to AC and WQ specimens.

**Figure 12:**
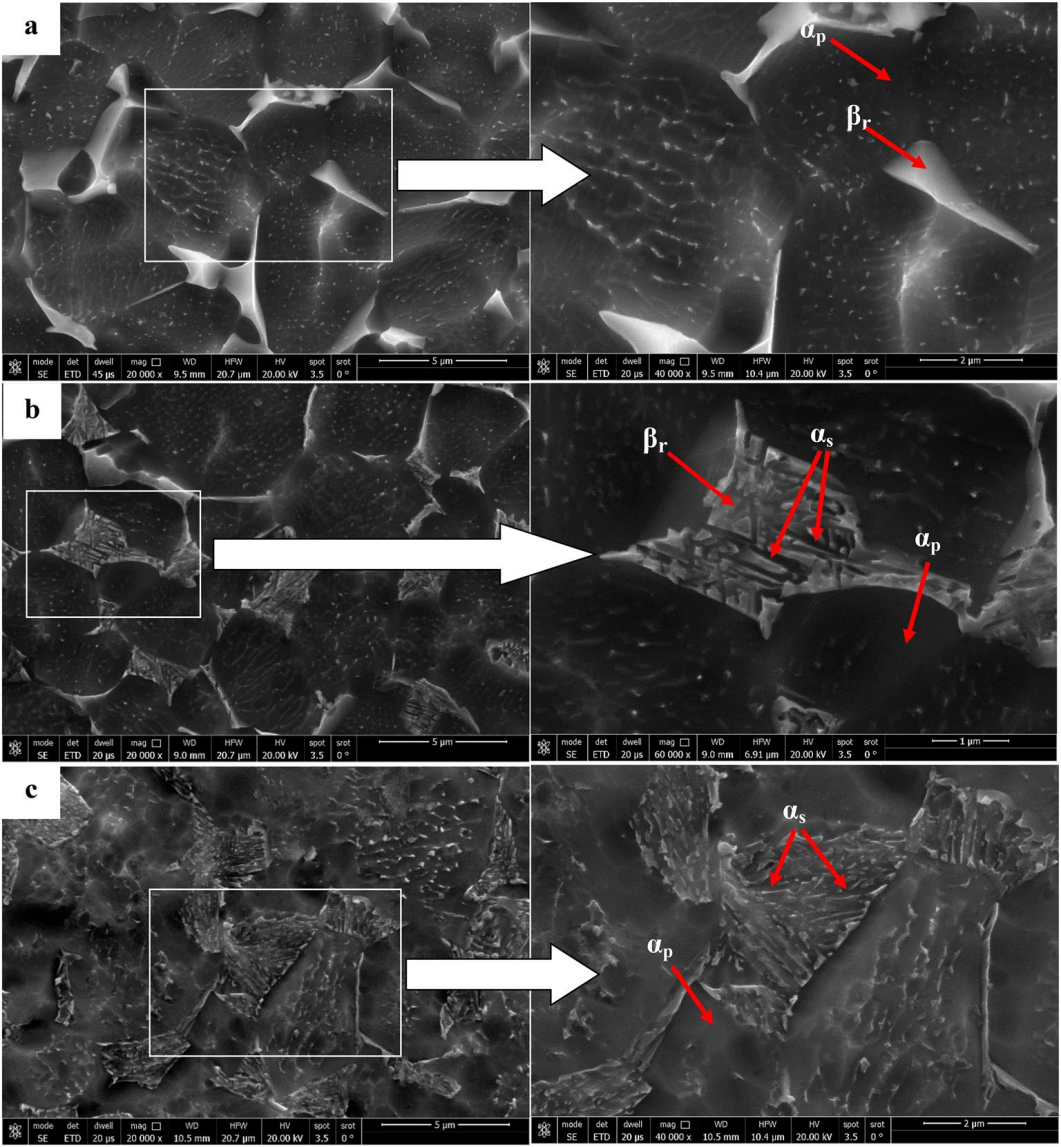
FESEM micrographs of aged specimens (**a**) FC + Aging, (**b**) AC + Aging, and (**c**) WQ + Aging.

**Figure 13:**
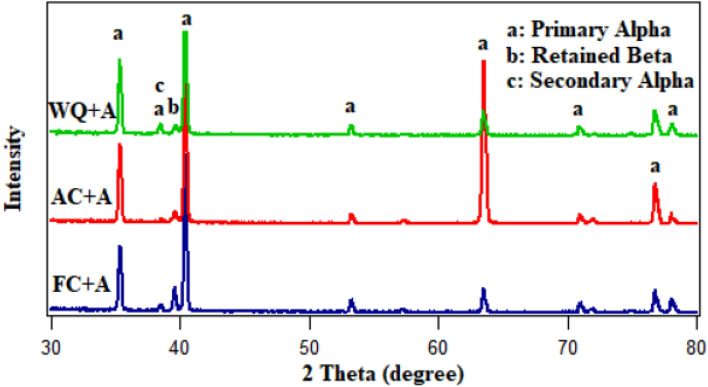
XRD pattern of FC + Aging, AC + Aging, and WQ + Aging specimens.

**Figure 14:**
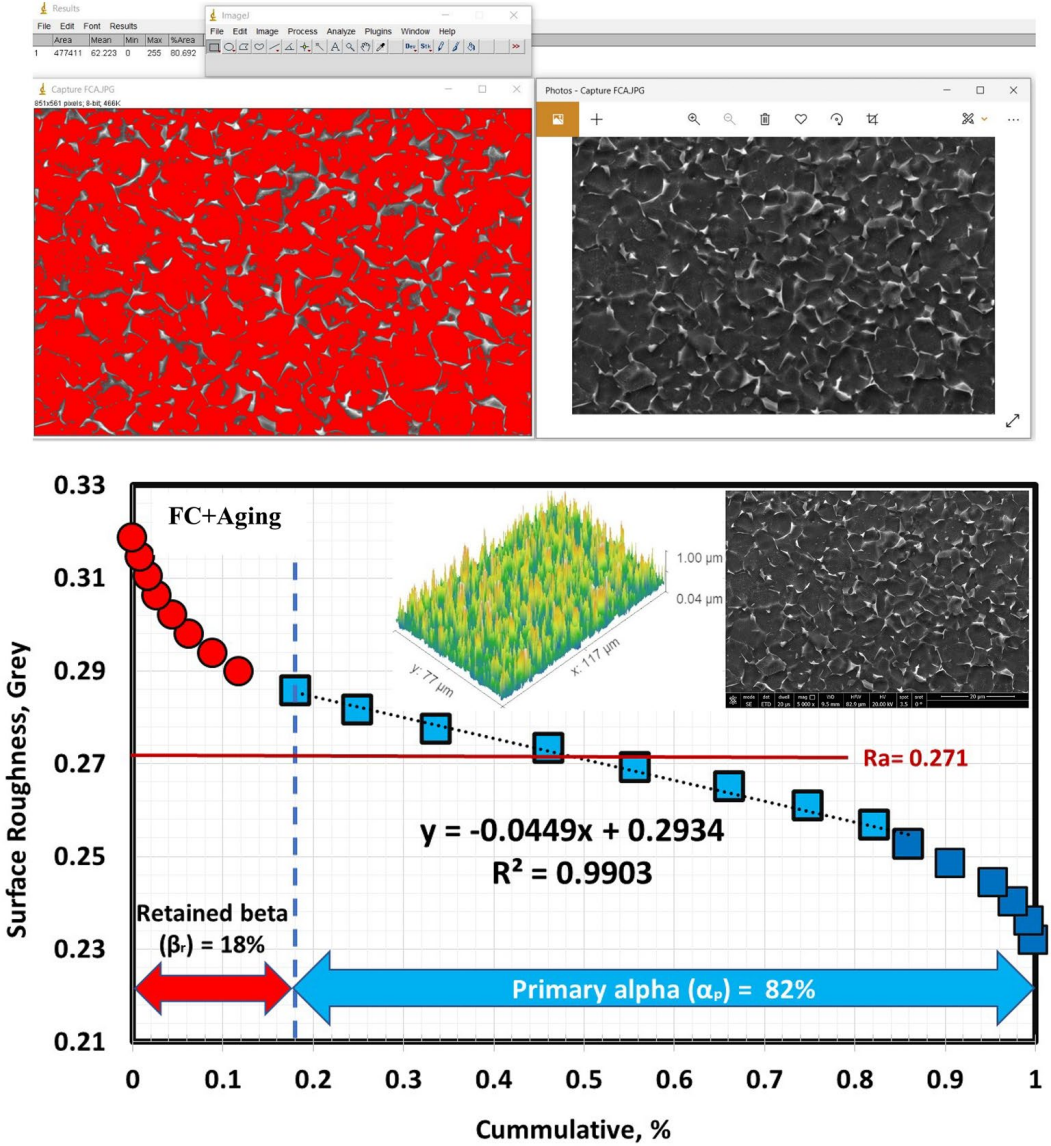
Volume fraction of α_p_-phase for FC + Aging specimen.

**Figure 15:**
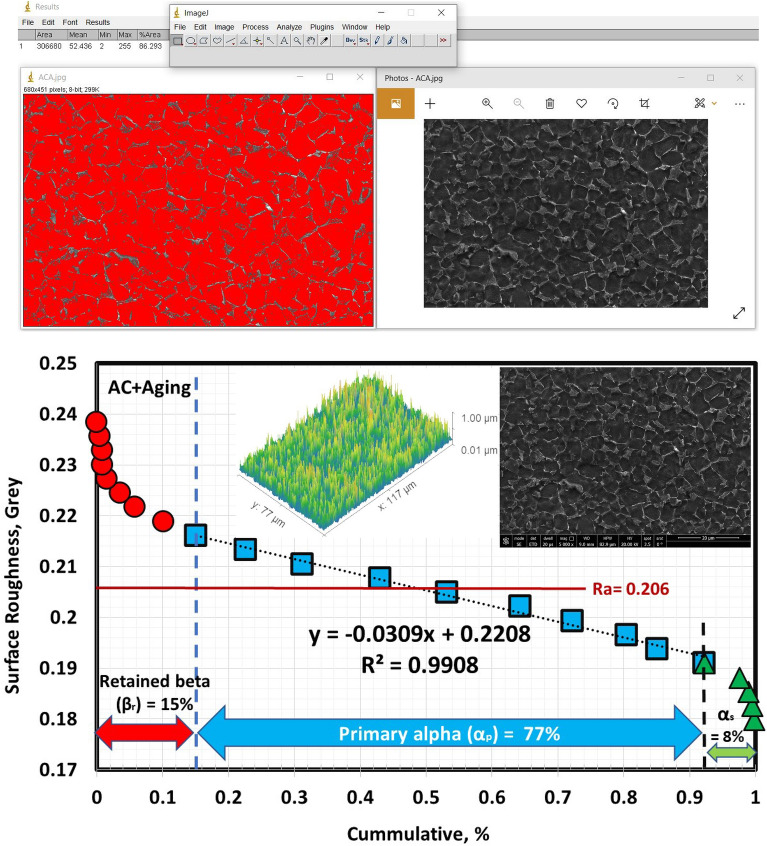
Volume fraction of α_p_-phase for AC + Aging specimen.

**Figure 16:**
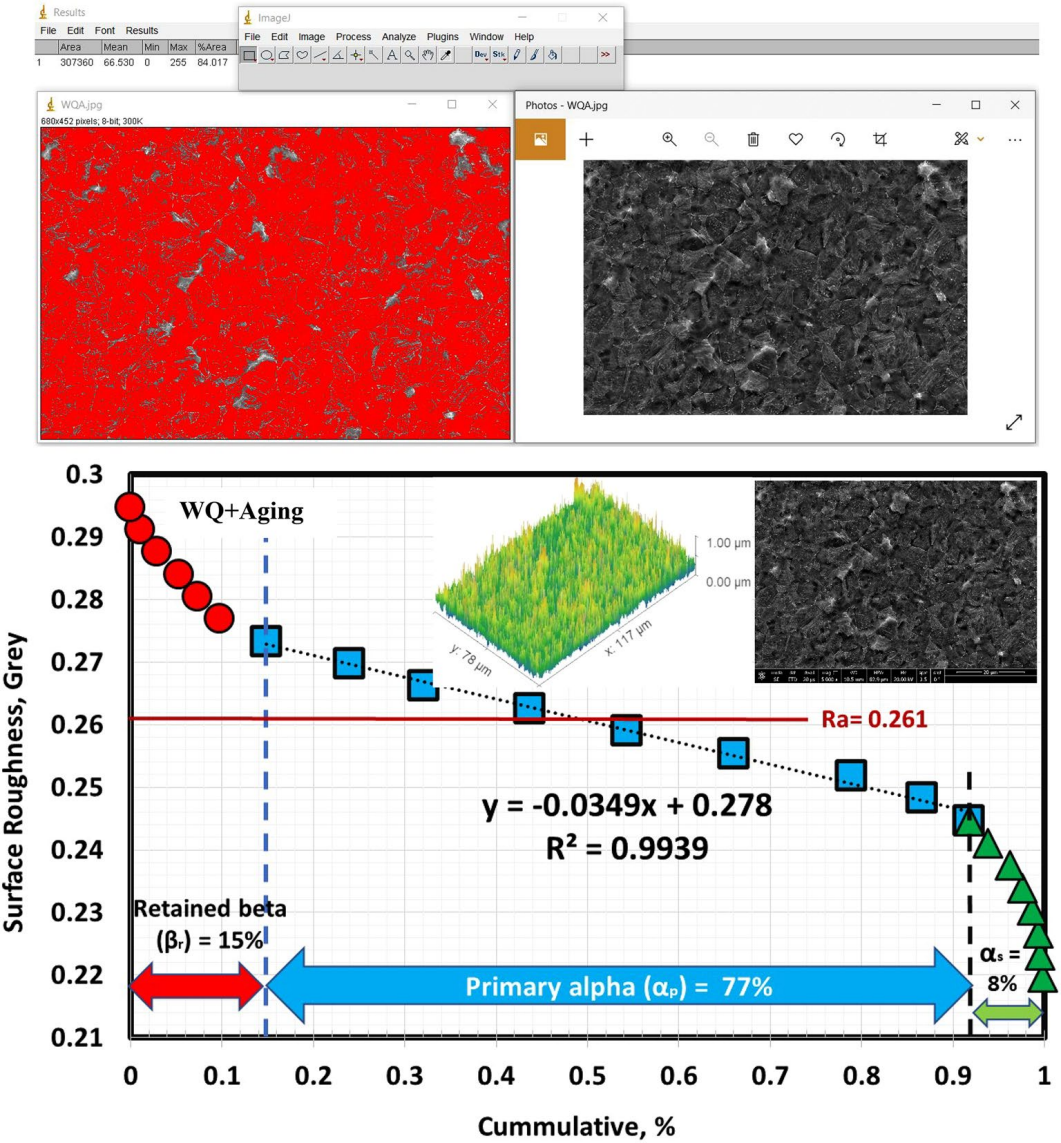
Volume fraction of α_p_-phase for WQ + Aging specimen.

The solution treatment and aging processes are among the most popular techniques for Ti6Al4V alloy^1^. Due to the solution treated at 925 °C being lower than T_β_ (980 °C), α_p_-phase in the original microstructure did not dissolve throughout solution treatment or aging. Figure 12 shows the microstructure after the aging process. The morphology of α and β-phases basically stayed the same throughout the aging process compared to the solution-treated one. However, this process results in variations in grain size and volume fraction of α_p_ and β_r_-phases, in addition to α_s_-phase precipitation inside β_r_-phase at all cooling rate conditions except FC. Figure 13 displays XRD patterns of the FC + Aging, AC + Aging and WQ + Aging specimens. The patterns confirmed the presence of the phases in the microstructure. Figures 14, 15, 16 show the volume fraction of α_p_ and β_r_-phases. 81% volume fraction of α_p_-phase was visible in the microstructure of the FC + Aging specimen (Fig. 14). However, as shown in Figs. 15 and 16, AC + Aging and WQ + Aging specimens achieved 86 and 84%, respectively.

### Hardness

**Figure 17:**
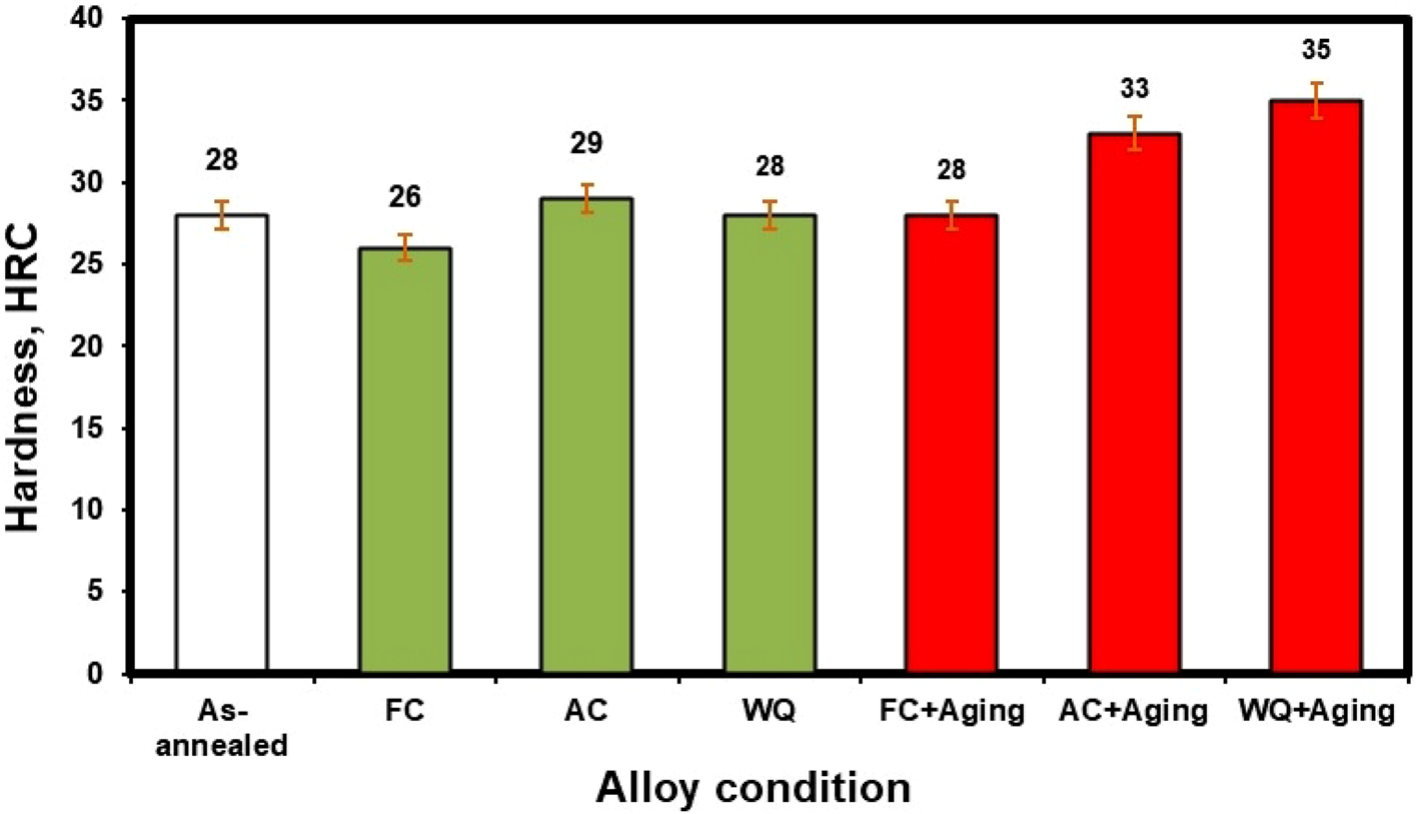
Hardness of the investigated Ti6Al4V alloy at various conditions.

Rockwell hardness tests were carried out to determine how the cooling rate and aging process affected the Ti6Al4V alloy in its as-annealed condition. Figure 17 displays the hardness fluctuation of as-annealed, solution-treated subjected to various cooling rates, and aged specimens. As-annealed specimen has a hardness of 28 HRC. After solution treatment, the hardness value of FC specimen (26 HRC) was relatively lower than that of the as-annealed specimen, which had the lowest hardness. This might be due to a high amount of α_p_-phase (81%). In contrast, WQ specimen showed a higher hardness (28 HRC) than FC specimen, perhaps because it included less α_p_-phase (only 68%) than the latter, and correspondingly the same hardness value of as- annealed specimen. Additionally, owing to the precipitation of α_s_-phase in β_r_-phase, AC specimen showed a maximum hardness of 29 HRC in contrast to the other cooling rates. From here it appears that there is a very small effect of solution treatment (925 °C) at different cooling rates on the hardness values.

Naturally, aging has an impact on hardness due to α_s_-phase precipitates occurring inside β_r_-phase. Both FC + Aging and AC + Aging samples had hardness values of 28 and 33 HRC, respectively. The maximum hardness value was 35 HRC for WQ + Aging specimen due to the presence of a high amount of β_r_ and α_s_-phase precipitation as well as a low amount of α_p_-phase in comparison to the others. This represents a rise in hardness values of roughly 25% as compared to the as-annealed specimen after WQ + Aging. Additionally, it is important to note that the three different cooling rates after solution treatment barely slightly affect hardness. However, the hardness varied significantly due to the aging process. For instance, the hardness of WQ + Aging increases by roughly 25% compared to WQ. This is because there is a significant amount of β_r_-phase resulting from α_p_-phase. The hardness of FC + Aging and AC + Aging also increased by 8 and 14%, respectively, compared to FC and AC. Compared to FC and AC specimens, the hardness variation in WQ specimen was more obvious. This is brought on by precipitation of the α_s_-phase and an increase in β_r_-phase volume percentage. However, for FC and AC specimens, a small amount of β_r_-phase and a large amount of α_p_-phase caused a slight increase in hardness during the aging process. Hardness is generally enhanced with the aging process, consistent with microstructure characteristics. In summary, aging considerably increases the hardness of WQ compared to AC and FC specimens.

## Tensile properties

**Figure 18:**
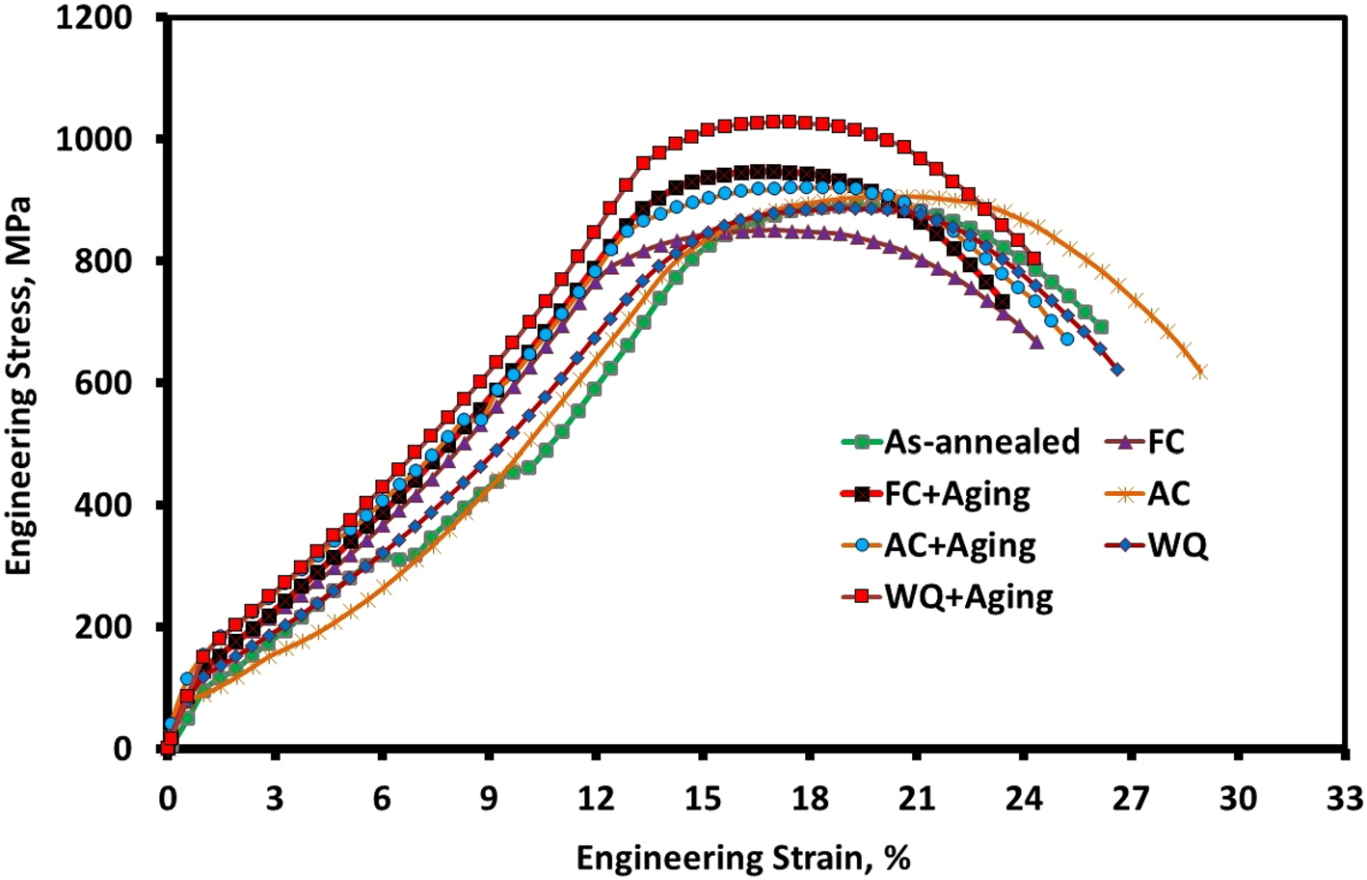
Engineering stress–strain curves of Ti6Al4V alloy at different conditions.

The engineering stress–strain curves of as-annealed, solution-treated at various cooling rates and aged specimens are shown in Fig. 18. The solution treatment at various cooling rates has an impact on the tensile properties of Ti6Al4V alloy, particularly the elongation values. Compared to as-annealed specimen, the elongation of FC, AC, and WQ specimens was 11 (+ 37.5%), 14 (+ 75%), and 12 (+ 50%), respectively. The ultimate tensile strength of AC specimen increased to 906 MPa (+ 1.68%) whereas it declined to 851 MPa (− 4.5%) for FC specimen and WQ specimen to 887 MPa (− 0.45%). The findings showed that WQ + Aging specimen had the highest ultimate tensile strength (1028 MPa), which was caused by its high amount of β_r_ with fine α_s_-phase. However, due to a significant volume fraction of α_p_-phase, FC specimen obtained the lowest ultimate tensile strength of 851 MPa. Due to α_s_-phase precipitates in β_r_-phase, the best balance of strength and elongation was discovered in WQ + Aging and AC + Aging specimens.

**Figure 19:**
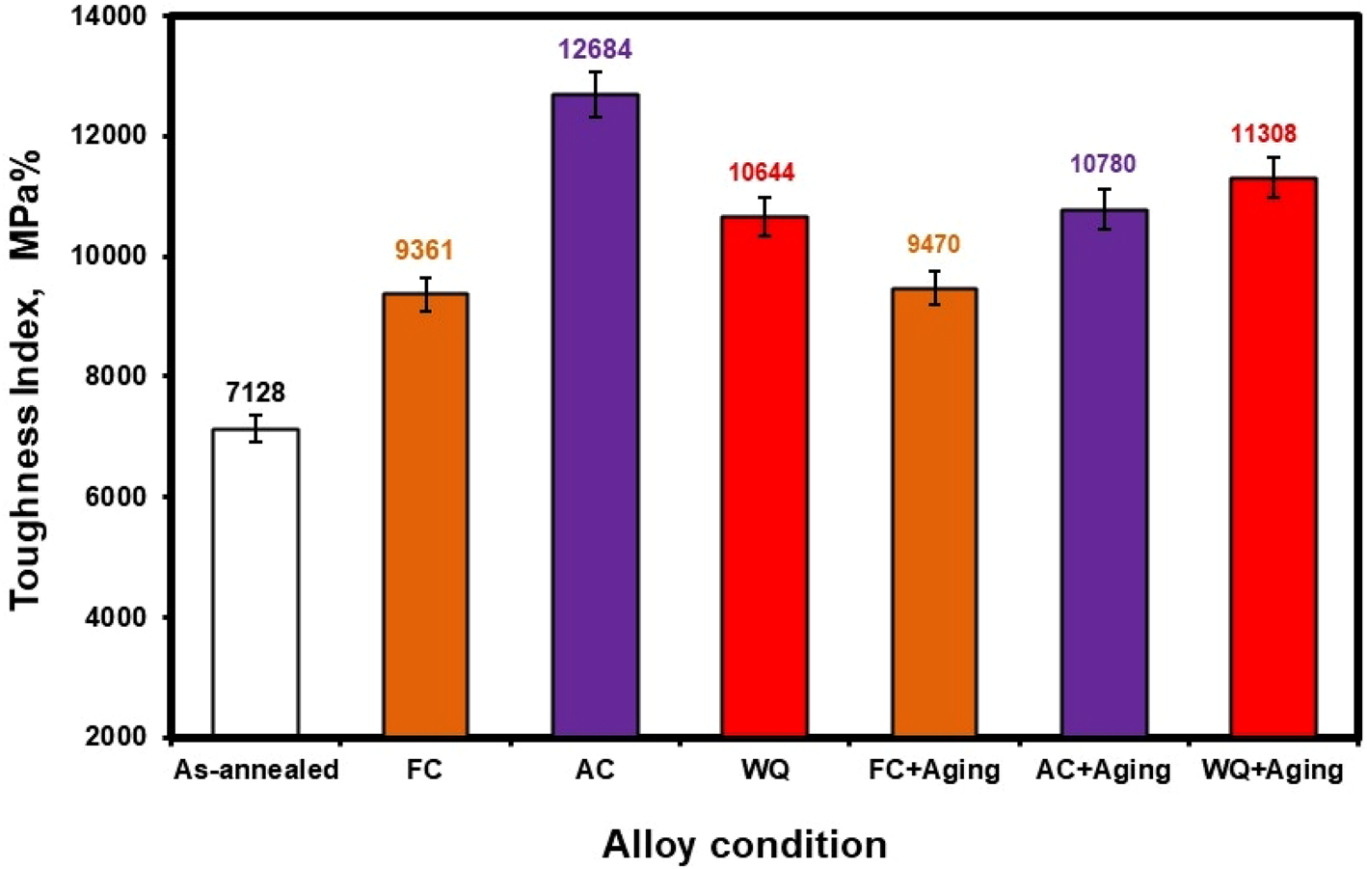
Toughness index of Ti6Al4V alloy at different conditions.

The toughness index (TI) of various conditions can be seen in Fig. 19. Using the formula ultimate strength x elongation, the IT was calculated. AC specimen had the best TI (12,684 MPa%), whereas the as-annealed specimen had the worst TI (7128 MPa%). As a result, when solution treated at 925 °C was applied, followed by AC, the TI increased to 78% as opposed to as-annealed specimen. Table 2 provides additional information regarding the hardness, tensile characteristics, and TI for different conditions.

**Table 2:** Hardness, tensile, and toughness index of Ti6Al4V alloy at different conditions.

Condition	Hardness, HRC	Yield Strength, MPa	Ultimate strength, MPa	Elongation, %	Toughness index, MPa%
As-annealed	28	775	891	8	7128
FC	26	749	851	11	9361
AC	29	792	906	14	12,684
WQ	28	762	887	12	10,644
FC + Aging	28	810	947	10	9470
AC + Aging	33	848	980	11	10,780
WQ + Aging	35	925	1028	11	11,308

**Figure 20:**
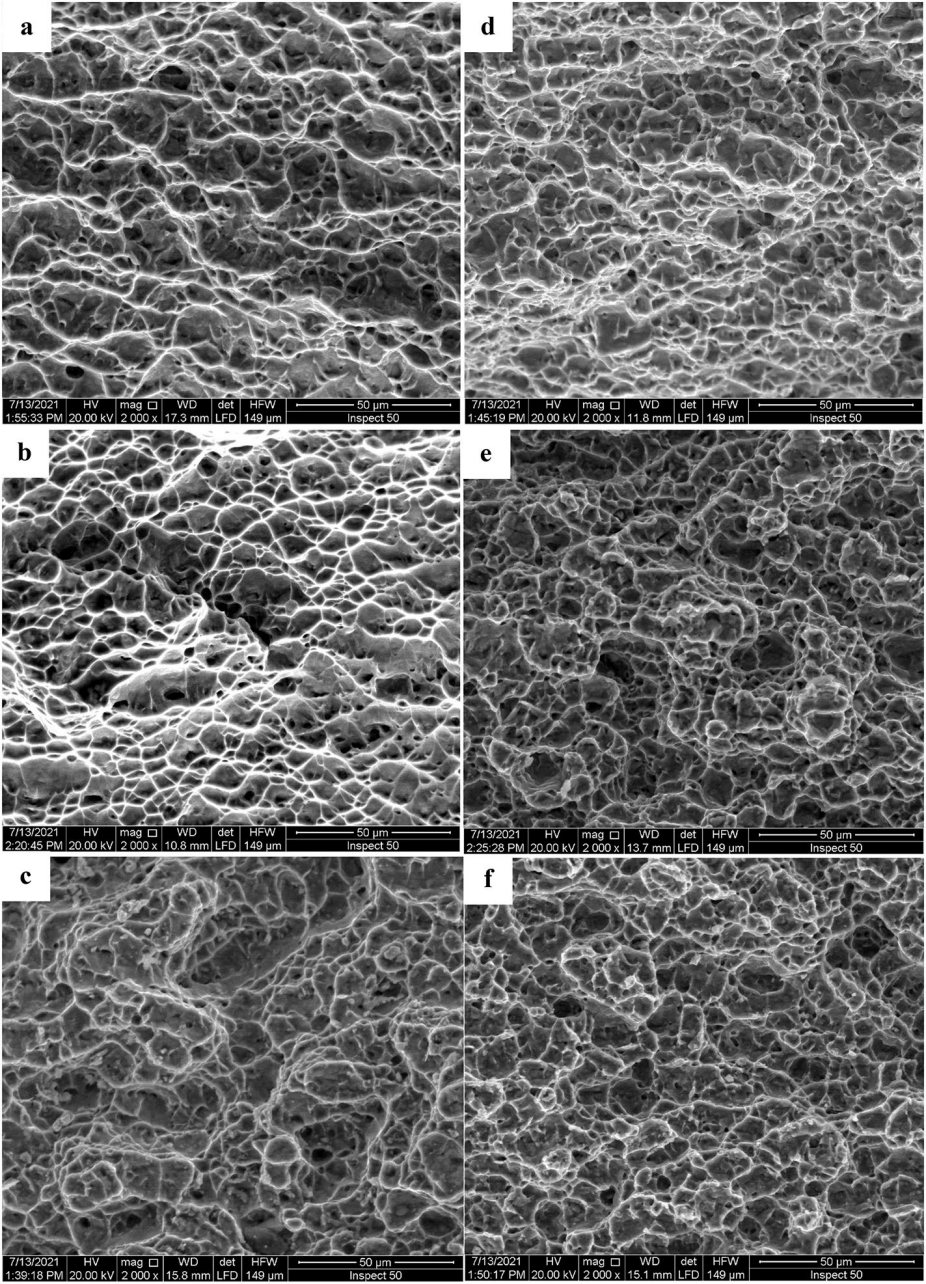
Fracture surface of tensile specimens of (**a**) FC, (**b**) AC, (**c**) WQ, (**d**) FC + Aging, (**e**) AC + Aging, and (**f**) WQ + Aging.

The fracture surface of tensile specimens for FC, AC, WQ, FC + Aging, AC + Aging and WQ + Aging specimens was examined using SEM, Fig. 20. Deep and homogenous dimples were observed on the fracture surface in all specimens and minor flat areas were found. Large shrinkage and plenty of deep and high amounts of ductile dimples were observed at the fracture surfaces of all specimens that prove good tensile elongation. The existing dimples can be classified into two categories: (i) narrow ductile dimples which may be induced by the separation of single α grain or β grain and (ii) wide ductile dimples which may be induced by the separation of several α grains^30^. Figure 20a–c shows that the width and depth of equiaxed ductile dimples increased with increasing the cooling rate. As the cooling rate increases, the tensile sample necks seriously before fracturing with big deep holes appearing on the surface. In addition, large shrinkage and plenty of deep and big equiaxed ductile dimples were noticed on WQ specimen's fracture surface, proving good elongation. The fracture surface of FC + Aging, AC + Aging and WQ + Aging specimens (Fig. 20d–f) consisted mainly of equiaxed ductile dimples and a very small percentage of flat areas. These dimples are relatively smaller and shallower than those observed in cases of FC, AC, and WQ specimens.

### Wear properties

**Figure 21:**
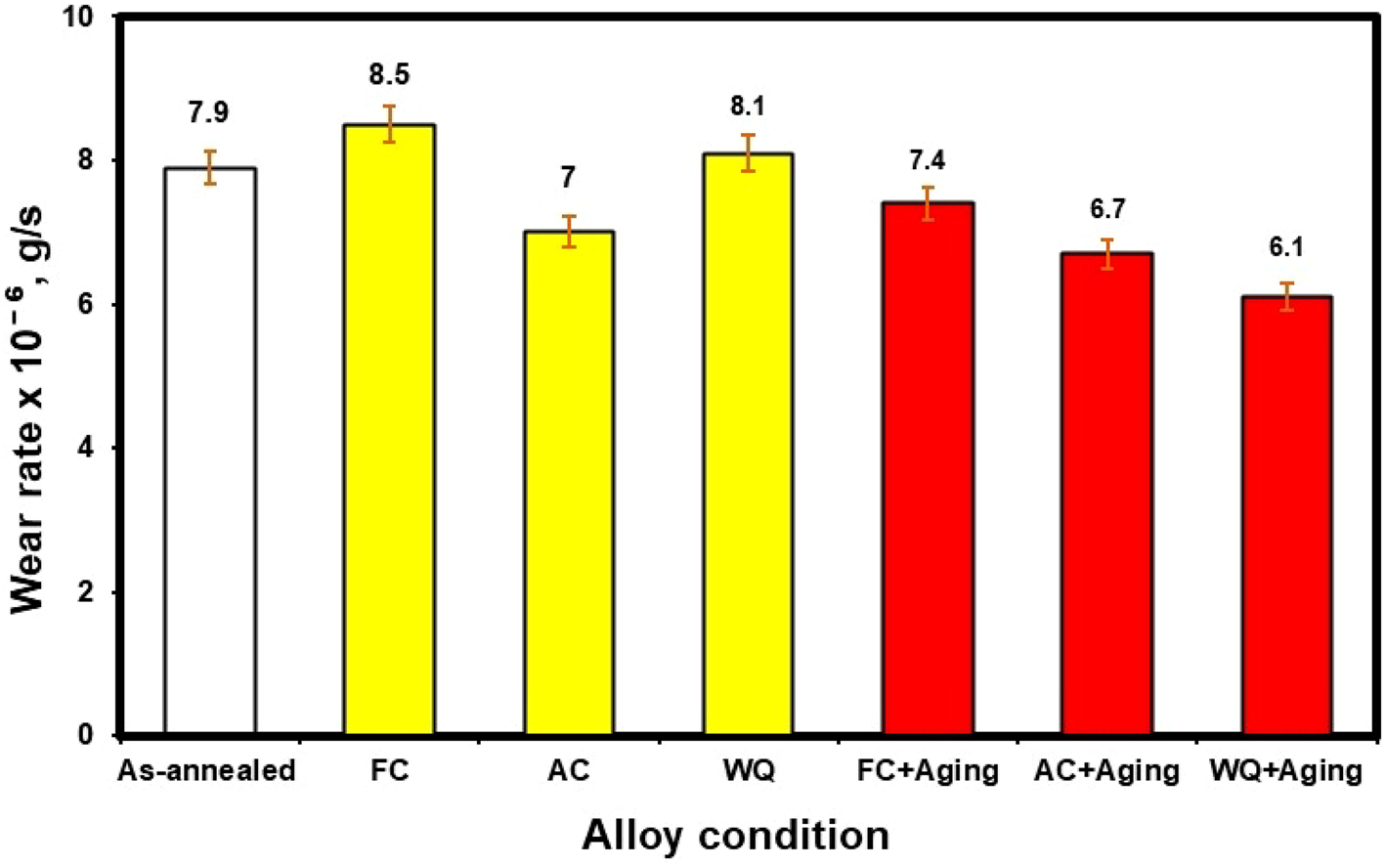
Wear rate of the investigated Ti6Al4V alloy at various conditions.

Figure 21 shows the wear rates for different conditions of as-annealed, FC, AC, WQ, FC + Aging, AC + Aging, and WQ + Aging. Each value represents the average of three specimens. According to Molinari et al.^31^, there are two basic causes for the poor tribological characteristics of titanium alloys: (1) low work-hardening threshold and plastic shearing resistance, (2) due to the high flash temperature brought on via friction during the wear process, surface oxide only offers minimal protection. Microstructure, temperature, and hardness are the primary elements affecting wear performance^32^. The as-annealed specimens showed a wear rate of 7.9 × 10^–6^ g/s. AC specimens obtained the lowest wear rate of 7 × 10^–6^ g/s. This may be due to existing of α_s_-phase (secondary α-platelets). While FC specimens revealed the highest wear rate of 8.5 × 10^–6^ g/s compared to as-annealed, AC and WQ specimens (the other conditions) due to the presence of a high amount of α_p_-phase (81%).

Archard's law states that the relationship between a material's wear rate and hardness is inverse^33^. Inferred from this is that a material's wear rate decreases with increasing hardness. The experimental sliding wear data showed a good correlation with Archard's law since the current conditions showed a considerable difference in hardness values. Due to their higher hardness compared to the other specimens, it was observed that the wear rate of WQ + Aging specimens increased. WQ + Aging specimens had the lowest reported wear rate as a result of their high hardness in comparison to all other conditions. Therefore, the hardness, or microstructural components, of the investigated specimens may be able to regulate the rate of wear.

Compared to a softer material, a harder one can typically maintain a thicker oxide coating more securely^34^. In light of this, the tougher WQ + Aging specimen might be able to firmly maintain an oxide layer that has a greater critical thickness before flaking off. There will be a change in wear rate when the oxide layer is removed, reformated, and thickened further. AC + Aging specimens displayed a wear rate (6.7 × 10^–6^ g/s). However, WQ specimens had the lowest wear rate (6.1 × 10^–6^ g/s) because they had a high percentage of β_r_-phase and a low percentage of α_p_ phase in comparison to the other specimens. When WQ and WQ + Aging specimens were compared, the specimens of WQ + Aging showed 25% reduction in wear rate as a result of the presence of a significant amount of β_r_-phase on account of α_p_-phase. In conclusion, compared to FC and AC specimens, the wear rate of WQ specimens is significantly increased by applying the aging process. This finding agrees with the study by Elshaer and Ibrahim^24^.

**Figure 22:**
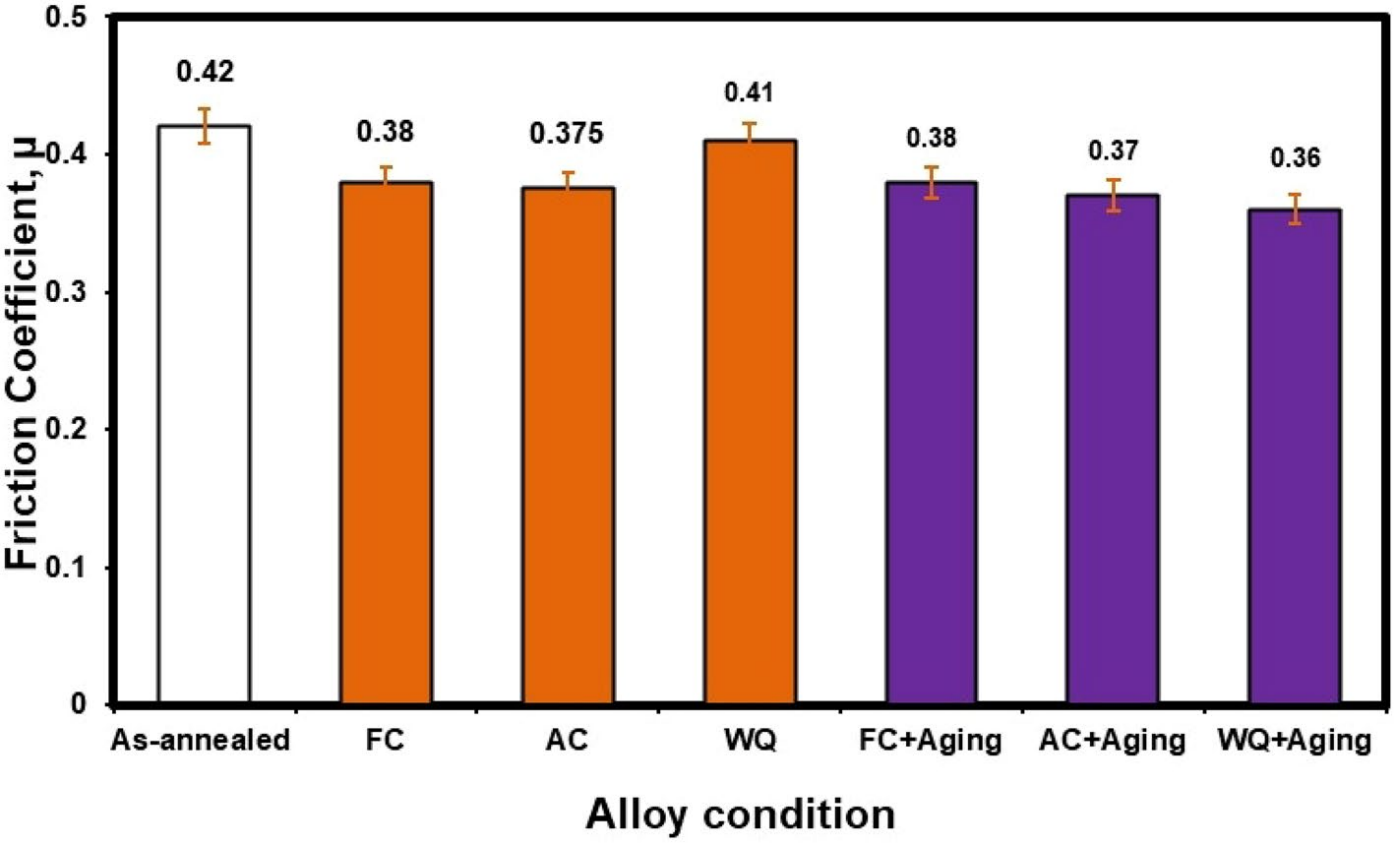
Friction coefficient of the investigated Ti6Al4V alloy at various conditions.

Figure 22 shows the friction coefficient of various conditions. It is clear that there is a minor difference in values of the friction coefficient between all conditions. Therefore, the heat treatment processes conducted on as-annealed specimens have no clear effect on the friction coefficient values.

**Figure 23:**
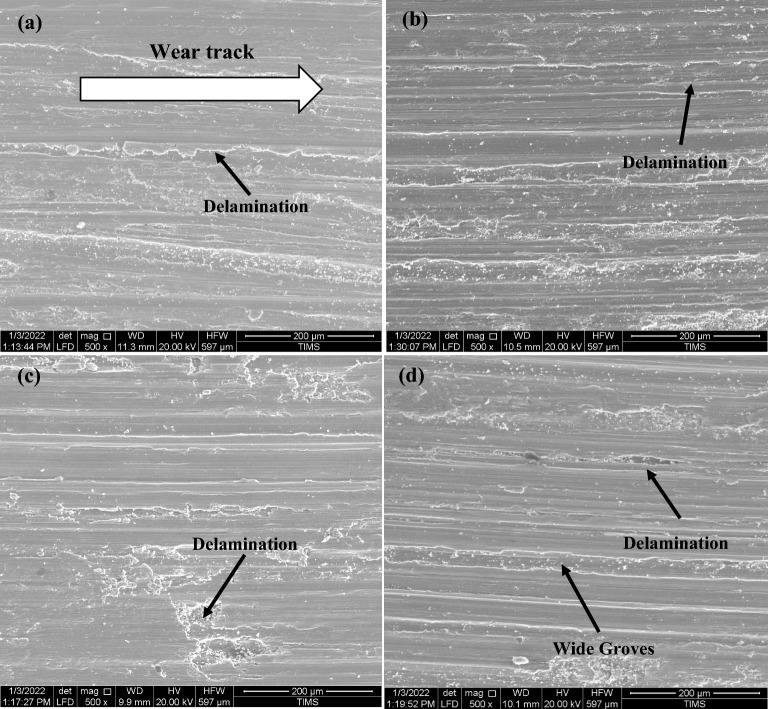
Worn surfaces of Ti6Al4V alloy at various conditions of (**a**) as-annealed, (**b**) FC, (**c**) AC, and (**d**) WQ.

**Figure 24:**
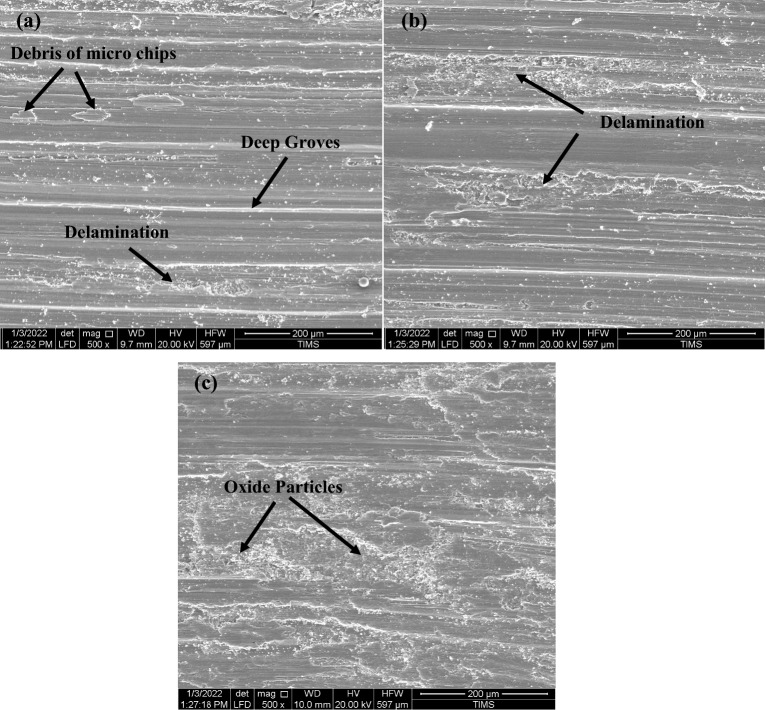
Worn surfaces of Ti6Al4V alloy at various conditions of (**a**) FC + Aging, (**b**) AC + Aging, and (**c**) WQ + Aging.

The morphological analysis of wear fragments was confirmed well with the above results. FESEM micrographs in Figs. 23 and 24 showed typically worn surface morphologies of some selected specimens tested of Ti6Al4V alloy at different conditions. Some evidence of abrasion wear was detected in all tested specimens. For various conditions, the wear track revealed a micro fragmentation mechanism. Also, for various conditions, Ti6Al4V alloy presented typical adhesive traces and abrasive furrows. It is clear that all conditions have delamination and groves except WQ + Aging due to higher hardness. Some debris and oxide particles were also observed at FC + Aging and WQ + Aging in Fig. 24 a, c.

## Conclusions

In the ongoing study on Ti6Al4V alloy, the effect of heat treatment processes on the microstructure, tensile and tribological properties of Ti6Al4V alloy was investigated. Some of the conclusions that can be drawn from this study include the following:

1. The microstructure is made up of primary α-phase (α_p_), secondary α-phase (α_s_) and retained β-phase (β_r_). When the specimens were solution treated (AC/WQ) and then aged, α_s_ precipitated inside β_r_.
2. Maximum hardness of 35 HRC was recorded for WQ + Aging specimen due to the existence of a high amount of β_r_-phase and precipitation of α_s_-phase. However, minimum hardness of 26 HRC was obtained for FC specimen.
3. The highest value for the elongation of AC specimen is 14%. However, the best ultimate tensile strength of 1028 MPa can be achieved for WQ + Aging specimen.
4. The perfect compromise between strength and elongation was found for WQ + Aging and AC + Aging specimens.
5. The maximum wear rate was obtained for WQ specimen. On the other hand, the minimum wear rate was given for WQ + Aging specimen due to its high hardness. The aging process that occurs after solution treatment can greatly enhance the wear property, and for WQ + Aging compared to WQ, this improvement can reach up to roughly 125%.

## Data Availability

All data generated or analyzed during this study are included in this published article.
